# Supplementary material for: SpatialDM for rapid identification of spatially co-expressed ligand–receptor and revealing cell–cell communication patterns
Source: Nat Commun. 2023 Jul 6;14:3995. doi: 10.1038/s41467-023-39608-w (PMC10325966; doi:10.1038/s41467-023-39608-w)
Supplement: Supplementary file 1 — Supplementary Information [file 41467_2023_39608_MOESM1_ESM.pdf]

Supplementary Information

SpatialDM for rapid identification of spatially co-expressed ligand-receptor and revealing cell-cell communication patterns

Contents

1 Supplementary Note: analytical null distribution of bi-variate Moran's R **1**

1.1 Univariate Moran's I 1

1.2 Bivariate Moran's global R 1

1.3 Bivariate Moran's local R 3

2 Supplementary Figure **5**

3 Supplementary References **13**

1 Supplementary Note: analytical null distribution of bi-variate Moran's R

1.1 Univariate Moran's I

Univariate Moran's I is established for hypothesis testing on the spatial autocorrelation of a single variable $\boldsymbol{x}$. Let $\boldsymbol{x} = (x_1, x_2, ..., x_n)$ be the vector of values over n locations, and $\bar{x}$ be the mean of $\boldsymbol{x}$. $W = w_{[ij]}$ is the spatial weight matrix (without normalization). The formula of the statistic takes the form in the following equation:

$$I = \frac{n}{\sum_{i=1}^{n}\sum_{j=1}^{n} w_{ij}} \frac{\sum_{i=1}^{n}\sum_{j=1}^{n} w_{ij}(x_i - \bar{x})(x_j - \bar{x})}{\sum_{i=1}^{n}(x_i - \bar{x})^2}$$

Null hypothesis assumes that

1) $\boldsymbol{x}$ is normally distributed

2) $\boldsymbol{x}$ is spatially randomly distributed

Under the assumption, the expectation and variance of univariate Moran's I are

$$E(I) = \frac{-1}{n-1}$$

$$Var(I) = \frac{n((n^2 - 3n + 3)S_1 - nS_2 + 3(\sum_{i=1}^{n}\sum_{j=1}^{n} w_{ij})^2}{(n-1)(n-2)(n-3)(\sum_{i=1}^{n}\sum_{j=1}^{n} w_{ij})^2} - (E(I))^2$$

$$S_1 = \frac{1}{2}\sum_{i=1}^{n}\sum_{j=1}^{n}(w_{ij} + w_{ji})^2, \quad S_2 = \sum_{i=1}^{n}(\sum_{j=1}^{n} w_{ij} + \sum_{j=1}^{n} w_{ji})^2$$

1.2 Bivariate Moran's global R

Here, in order to distinguish the univariate auto-correlation, we use the symbol R to indicate spatial correlation. Specifically, the multivariate Moran's R indicates the spatial correlation between one variable and another variable in neighbouring regions. Here we consider the bivariate case. Let $\boldsymbol{X} = (X_1, X_2, ..., X_n)$ and $\boldsymbol{Y} = (Y_1, Y_2, ..., Y_n)$ be the two variables of interest. The formula of the statistic takes the form in the following equation:

$$R = \frac{n}{\sum_{i=1}^{n}\sum_{j=1}^{n} w_{i,j}} \frac{\sum_{i=1}^{n}\sum_{j=1}^{n} w_{ij}(X_i - \bar{X})(Y_j - \bar{Y})}{\sqrt{\sum_{i=1}^{n}(X_i - \bar{X})^2}\sqrt{\sum_{i=1}^{n}(Y_i - \bar{Y})^2}} \tag{1}$$

The null hypothesis assumes that

1) $\boldsymbol{X}$ and $\boldsymbol{Y}$ are normally distributed: $\boldsymbol{X} \sim N(0, \sigma_1^2 I), \boldsymbol{Y} \sim N(0, \sigma_2^2 I)$.

2) $\boldsymbol{X}$ and $\boldsymbol{Y}$ are is spatially randomly distributed.

The expectation and variance of the test statistic need to be derived under the null hypothesis to conduct the z-score test. Note, in Eq. (1), we can further absorb the normalisation term of $\mathbf{W}$ to simplify the notation. However, the de-mean operations over $\boldsymbol{X}$ and $\boldsymbol{Y}$ cannot be absorbed for simplification, as it will break the i.i.d. assumption. Instead, we have to introduce a centring matrix below.

Denote $\mathbf{H}$ as the centring matrix, $\mathbf{H} = I - \frac{1}{n}\mathbf{1}\mathbf{1}^T$, then Eq. (1) can be rewritten in matrix form:

$$
\begin{aligned}
\mathbf{R} &= \frac{n}{\sum_{i=1}^{n}\sum_{j=1}^{n} w_{i,j}} \frac{(\mathbf{HX})^T\mathbf{WHY}}{\sqrt{(\mathbf{HX})^T\mathbf{HX}}\sqrt{(\mathbf{HY})^T\mathbf{HY}}} \\
&= \frac{n}{\sum_{i=1}^{n}\sum_{j=1}^{n} w_{i,j}} \frac{\mathbf{X}^T\mathbf{HWHY}}{\sqrt{\mathbf{X}^T\mathbf{HHX}}\sqrt{\mathbf{Y}^T\mathbf{HHY}}} \\
&= \frac{n}{\sum_{i=1}^{n}\sum_{j=1}^{n} w_{i,j}} \frac{\mathbf{X}^T\mathbf{HWHY}}{\sqrt{\mathbf{X}^T\mathbf{HXYY}^T\mathbf{HY}}}
\end{aligned} \tag{2}
$$

Since $\mathbf{H}$ is symmetric, diagonalizing $\mathbf{H}$ will give

$$
\mathbf{H} = \mathbf{P}^T Diag(1, 1, , , , 1, 0)\mathbf{P},
$$

where $\mathbf{P}$ is an orthogonal matrix.

Apply the same orthogonal transformation to the numerator in Eq. (2):

$$
\begin{aligned}
\mathbf{P^THWHP} &= \mathbf{P^THPP^TWPP^THP} \\
&= Diag(1, 1, , , , 1, 0)\mathbf{P^TWP}Diag(1, 1, , , , 1, 0) = \mathbf{P^TWP_{n-1}},
\end{aligned}
$$

where $\mathbf{P^TWP_{n-1}}$ denotes the submatrix of $\mathbf{P^TWP}$ consisting of its first $(n-1)$ -th rows and columns. Hence it is also symmetric. Suppose that further diagonalising yields

$$
\mathbf{P^TWP_{n-1}} = \mathbf{Q_1^T} Diag(\lambda_1, \lambda_2, ..., \lambda_{n-1})\mathbf{Q_1}, \tag{3}
$$

where $\lambda_1, \lambda_2, ..., \lambda_{n-1}$ are the $n-1$ eigenvalues of the matrix $\mathbf{P^TWP_{n-1}}$ and $\mathbf{Q_1}$ is orthogonal. Let

$$
\mathbf{Q} = \begin{pmatrix} Q_1 & 0 \\ 0 & 1 \end{pmatrix} \tag{4}
$$

$$
\mathbf{V} = \mathbf{PQ} \tag{5}
$$

Orthogonality of P and Q ensures that V is also orthogonal. Observe that V can diagonalise both HWH and H:

$$
\mathbf{V^THWHV} = \mathbf{Q^TP^THWHPQ} = Diag(\lambda_1, \lambda_2, ..., \lambda_{n-1}, 0) \tag{6}
$$

$$
\mathbf{V^THV} = \mathbf{Q^T} Diag(1, 1, , , , 1, 0)\mathbf{Q} = Diag(1, 1, , , , 1, 0) \tag{7}
$$

Substitute Eq. (6) and (7) into (2), bivaraite Moran's R can be rewritten into

$$
R = \frac{n}{\sum_{i=1}^{n}\sum_{j=1}^{n} w_{i,j}} \frac{\mathbf{X^TV}Diag(\lambda_1, \lambda_2, ..., \lambda_{n-1}, 0)\mathbf{V^TY}}{\sqrt{\mathbf{X^TV}Diag(1, 1, , , , 1, 0)\mathbf{V^TX}}\sqrt{\mathbf{Y^TV}Diag(1, 1, , , , 1, 0)\mathbf{V^TY}}} \tag{8}
$$

Let $\mathbf{V^TX} = (a_1, a_2, , , , a_n)$ and $\mathbf{V^TY} = (b_1, b_2, , , , b_n)$. Since $\mathbf{V}$ is orthogonal, $\mathbf{V^TX}$ and $\mathbf{V^TY}$ still follows the same normal distribution, that is,

$$
\mathbf{V^TX} \sim N(0, \sigma_1^2 I) \tag{9}
$$

$$
\mathbf{V^TY} \sim N(0, \sigma_2^2 I) \tag{10}
$$

or equivalently, for any $i = 1, 2, ..., n$, $a_i \sim N(0, \sigma_1^2)$, $b_i \sim N(0, \sigma_2^2)$. Then R can be expressed into a function of product of normal random variables:

$$
R = \frac{n}{\sum_{i=1}^{n}\sum_{j=1}^{n} w_{i,j}} \frac{\sum_{i=1}^{n-1} \lambda_i a_i b_i}{\sqrt{\sum_{i=1}^{n-1} a_i^2}\sqrt{\sum_{i=1}^{n-1} b_i^2}} \tag{11}
$$

Assuming that $E(R^p) = \frac{E(numerator^p)}{E(denominator^p)}$, it suffices to compute the moments of numerator and denominator respectively.
By the assumption,

$$E(a_i)E(b_i) = 0 \tag{12}$$

$$E(a_i^2)E(b_i^2) = \sigma_1^2\sigma_2^2 \tag{13}$$

$$E(a_i a_j b_i b_j) = 0 \tag{14}$$

Hence,

$$E(numerator) = \sum_{i=1}^{n-1} \lambda_i E(a_i)E(b_i) = 0 \tag{15}$$

$$E(numerator^2) = \sum_{i=1}^{n-1} \lambda_i^2 E(a_i^2)E(b_i^2) + 2\sum_{i \neq j} \lambda_i\lambda_j E(a_i a_j b_i b_j) \tag{16}$$

$$E(denominator^2) = \sum_{i=1}^{n-1} E(a_i^2) \sum_{i=1}^{n-1} E(b_i^2) = (n-1)^2\sigma_1^2\sigma_2^2 \tag{17}$$

Combining (15),(16) and (17),

$$E(R) = 0 \tag{18}$$

$$Var(R) = E(R^2) = \frac{n^2}{(\sum_{i=1}^{n}\sum_{j=1}^{n} w_{i,j})^2} \frac{\sum_{i=1}^{n-1} \lambda_i^2}{(n-1)^2} \tag{19}$$

Recall (6), it is equivalent to $\mathbf{HWH} = \mathbf{V}Diag(\lambda_1, \lambda_2, ..., \lambda_{n-1}, 0)\mathbf{V^T}$. Thus, $\lambda_1, \lambda_2, ..., \lambda_{n-1}$ are also eigenvalues of $\mathbf{HWH}$. By the property of trace and eigenvalues,

$$\sum_{i-1}^{n-1} \lambda_i^2 = tr((\mathbf{HWH})^2) \tag{20}$$

Substitute (20) into and represent the trace using the elements of W, the variance of bivariate Moran's R can be expressed in the form of function of matrix W:

$$Var(R) = \frac{n^2 \sum_{i=1}^{n}\sum_{j=1}^{n} w_{ij}w_{ji} - 2n(\sum_{i=1}^{n}(\sum_{j=1}^{n} w_{ij}\sum_{j=1}^{n} w_{ji}) + (\sum_{i=1}^{n}\sum_{j=1}^{n} w_{ij})^2}{n^2(n-1)^2} \tag{21}$$

1.3 Bivariate Moran's local R

Local moran's R at the i-th spot takes the form in the following equation:

$$R_i = (x_i - \bar{x})\sum_{j=1}^{n} w_{ij}(y_j - \bar{y}) + (y_i - \bar{y})\sum_{j=1}^{n} w_{ij}(x_j - \bar{x}) \tag{22}$$

Since

$$x_i - \bar{x} = \frac{n-1}{n}x_i - \frac{1}{n}\sum_{j=1, j\neq i}^{n} x_j \tag{23}$$

and each pair of x_i and x_j for any i and j are independent, for any i,

$$x_i - \bar{x} \sim N(0, \frac{n-1}{n}\sigma_1^2 I) \tag{24}$$

Similarly,

$$y_i - \bar{y} \sim N(0, \frac{n-1}{n}\sigma_2^2 I) \tag{25}$$

Hence,

$$E(R_i) = E(x_i - \bar{x})\sum_{j=1}^{n} w_{ij}E(y_j - \bar{y}) + E(y_i - \bar{y})\sum_{j=1}^{n} w_{ij}E(x_j - \bar{x}) = 0 \tag{26}$$

$$\begin{aligned} Var((x_i - \bar{x})w_{ij}(y_j - \bar{y})) &= w_{ij}^2(E((x_i - \bar{x})^2(y_j - \bar{y})^2) - (E((x_i - \bar{x})(y_j - \bar{y}))^2)) \\ &= w_{ij}^2 E((x_i - \bar{x})^2(y_j - \bar{y})^2) = w_{ij}^2 E((x_i - \bar{x})^2)E((y_j - \bar{y})^2) \\ &= w_{ij}^2 \frac{(n-1)^2}{n^2}\sigma_1^2\sigma_2^2 \end{aligned} \tag{27}$$

$$\begin{aligned} Cov((x_i - \bar{x})w_{ij}(y_j - \bar{y}), (y_i - \bar{y})w_{ik}(x_k - \bar{x})) &= E((x_i - \bar{x})w_{ij}(y_j - \bar{y})(y_i - \bar{y})w_{ik}(x_k - \bar{x})) \\ &\quad - E((x_i - \bar{x})w_{ij}(y_j - \bar{y}))E((y_i - \bar{y})w_{ik}(x_k - \bar{x})) \\ &= E((x_i - \bar{x})w_{ij}(y_j - \bar{y})(y_i - \bar{y})w_{ik}(x_k - \bar{x})) \\ &\neq 0 \text{ if and only if i = j = k} \end{aligned} \tag{28}$$

$$\begin{aligned} Cov((x_i - \bar{x})w_{ij}(y_j - \bar{y}), (x_i - \bar{x})w_{ik}(y_k - \bar{y})) &= E((x_i - \bar{x})w_{ij}(y_j - \bar{y})(x_i - \bar{x})w_{ik}(y_k - \bar{y})) \\ &\quad - E((x_i - \bar{x})w_{ij}(y_j - \bar{y}))E((x_i - \bar{x})w_{ik}(y_k - \bar{y})) \\ &= 0 \end{aligned} \tag{29}$$

$$\begin{aligned} Cov((y_i - \bar{y})w_{ij}(x_j - \bar{x}), (y_i - \bar{y})w_{ik}(x_k - \bar{x})) &= E((y_i - \bar{y})w_{ij}(x_j - \bar{x})(y_i - \bar{y})w_{ik}(x_k - \bar{x})) \\ &\quad - E((y_i - \bar{y})w_{ij}(x_j - \bar{x}))E((y_i - \bar{y})w_{ik}(x_k - \bar{x})) \\ &= 0 \end{aligned} \tag{30}$$

Therefore,

$$\begin{aligned} Var(R_i) &= \sum_{j=1}^{n} Var((x_i - \bar{x})w_{ij}(y_j - \bar{y})) + \sum_{j=1}^{n} Var((y_i - \bar{y})w_{ij}(x_j - \bar{x})) \\ &\quad + 2Cov((x_i - \bar{x})w_{ii}(y_i - \bar{y}), (y_i - \bar{y})w_{ii}(x_i - \bar{x}) \\ &= 2\frac{(n-1)^2}{n^2}\sigma_1^2\sigma_2^2\sum_{j=1}^{n} w_{ij}^2 + 2\frac{(n-1)^2}{n^2}\sigma_1^2\sigma_2^2 w_{ii}^2 \end{aligned} \tag{31}$$

2 Supplementary Figure

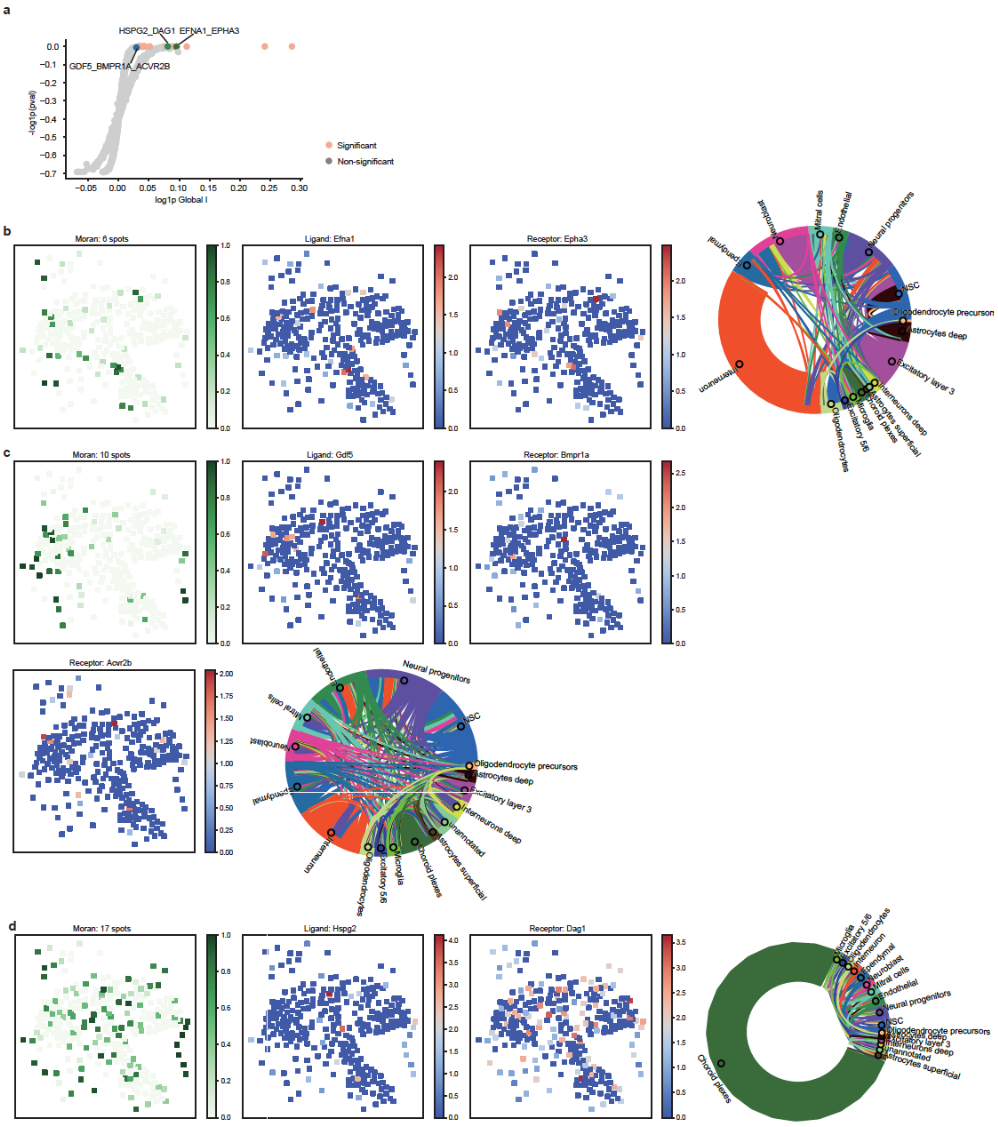

(a)

Supplementary Figure 1: **Detecting spatial LRI in mouse subventricular zone (SVZ).** We demonstrated the broad applicability of SpatialDM from Next-Generation Sequencing data to Fluorescent In Situ Hybridization (FISH) data. SVZ, located along the walls of the brain lateral ventricles, is the birthplace for neural stem cells throughout life. Many LRIs have been identified as essential to the neurogenic niche, including mitogenic signals like fibroblast growth factor 2 (FGF-2) and epidermal growth factor (EGF), neurogenic signals like BMP and Shh, as well as membrane-bound signals like Notch and Eph [1, 4]. All statistical tests here are one-sided. (a) From the limited number of significant interactions (FDR > 0.1) due to low sequencing coverage of FISH-based sequencing, we found (b) *EFNA1_EPHA3* enriched in neural progenitors, neuroblast and neural NSCs [4], (c) GDF5 signalling to *BMPR1A_ACVR2B* complex from various cell types, in particular NSC and neural progenitors [3], and (d) *HSPG2_DAG1* transmitted between adjacent choroid plexes cells [2], etc. In the Moran p-value spatial plots, dot colour indicates 1 - p-values (of all ranges), the selected number of spots refers to only spots with the uncorrected $p < 0.1$. The chord diagram visualizes the dominant cell types where edge numbers correspond to weighted cell type composition on selected spots and the edge colour indicates sender cell types. All statistical results are based on one-sided tests without adjustment.

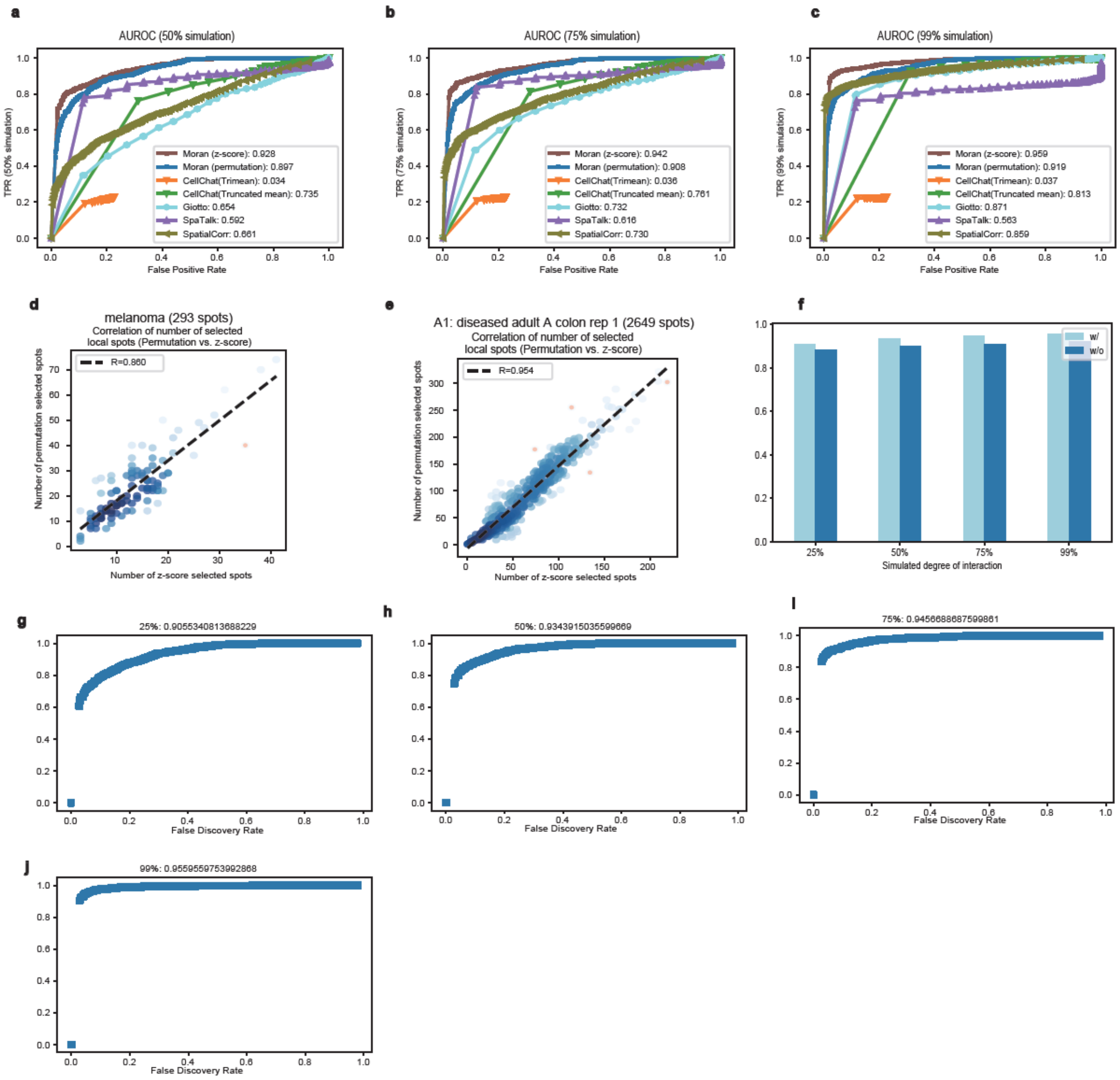

(a)

Supplementary Figure 2: **SpatialDM achieves high accuracy and reproducibility in real data and simulations.** (a-c) ROC under the 50% (a), 75% (b), 99% (c) degrees of interaction simulation scenario. AUROC for each method is labelled in the legend. Source data are provided as a Source Data file. (d-e) Pearson correlations (two-sided) of the numbers of local selected spots ($p < 0.1$) between z-score approach and permutation approach, in the melanoma dataset (d, $p < 1e{-}16$) and the intestine dataset (e, $p < 1e{-}16$). Source data are provided as a Source Data file. (f) AUROC comparison for global Moran permutation tests with (0.17 weight) vs. without considering ligand auto-correlation in each simulation scenario. (g-j) ROC under the 50% (a), 75% (b), 99% (c) degrees of interaction after considering ligand auto-correlation.

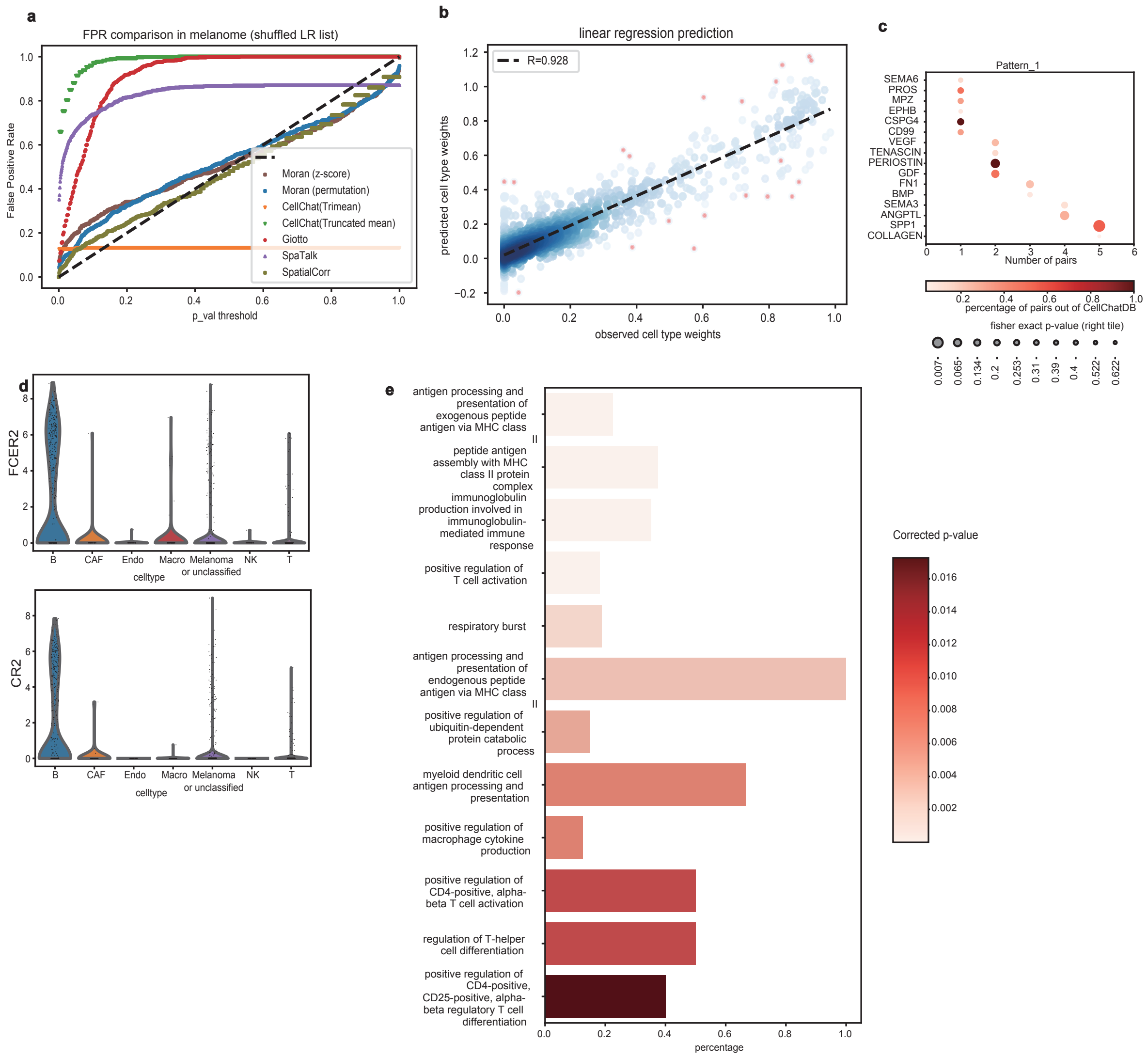

(a)

Supplementary Figure 3: **SpatialDM detects oncology-relevant interactions in the melanoma data.** (a) FPR comparison of SpatialDM with CellChat, Giotto, SpaTalk, and SpatialCorr in the melanoma dataset but with shuffled LR pairs. All tests are one-sided. Only SpatialDM (both z-score and permutation approaches) calibrates with the null distribution along with the diagonal line. (b) Cell type prediction based on local Moran p-values. The whole dataset was used for fitting the linear regression model and for prediction. A Pearson coefficient $R = 0.928$ was observed. Source data are provided as a Source Data file. (c) Pathway enrichment result for pattern 3 interactions (one-sided). Source data are provided as a Source Data file. (d) FCER2 and CR2 expression across different cell types in the scRNA dataset. (e) Gene Ontology enrichment of the 3500 genes that are up-regulated in the CD23 hot spots (one-sided Fisher's Exact Test, Benjamini-Hochberg corrected. Source data are provided as a Source Data file.).

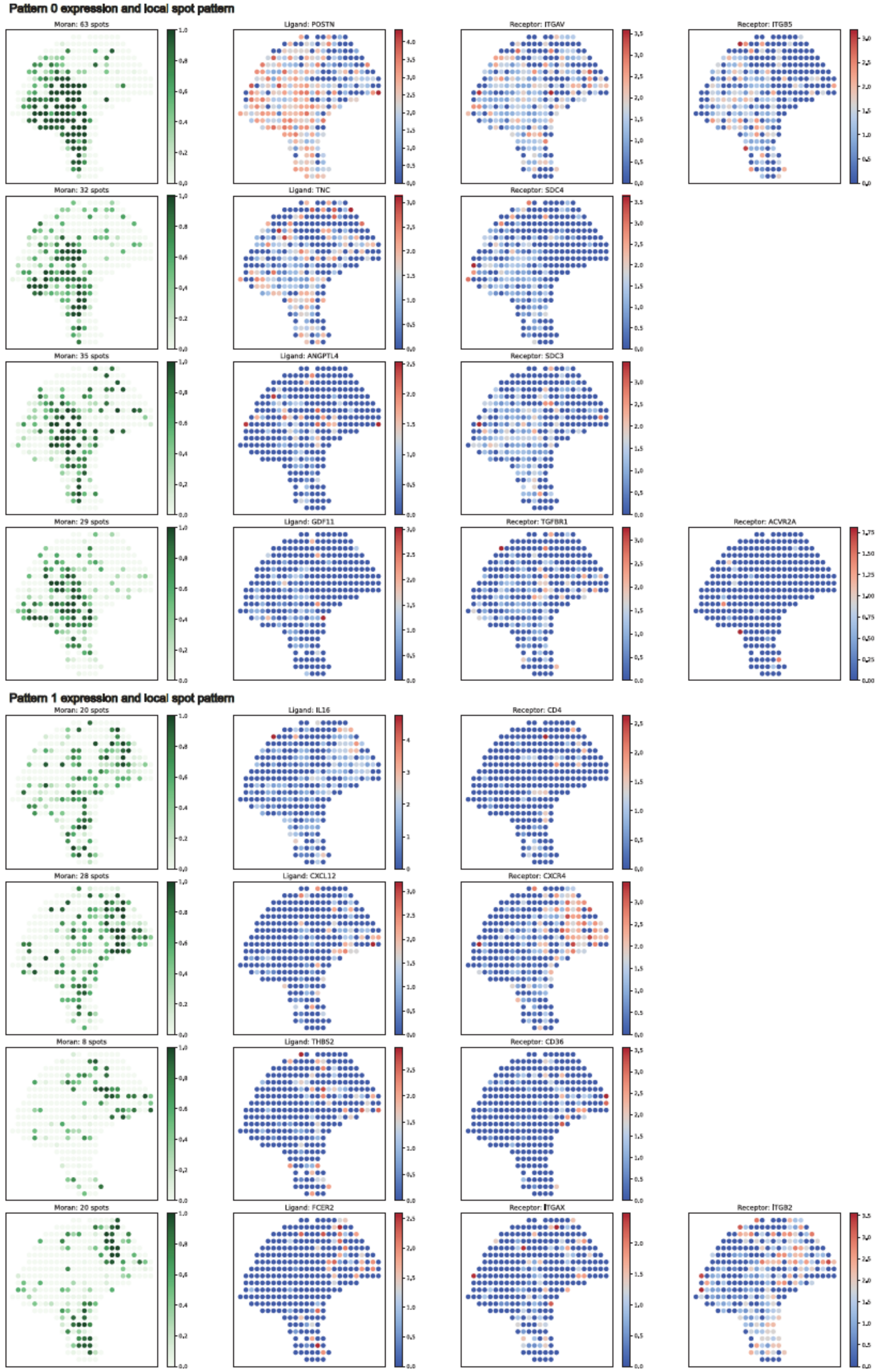

(a)

Supplementary Figure 4: **4 exemplar interactions belonging to pattern 0 (Upper panel) and pattern 1 (Lower panel), respectively**. The local Moran spots (permutation, $p < 0.1$, one-sided), and their corresponding LR expression were visualised. Local p of all ranges are displayed.

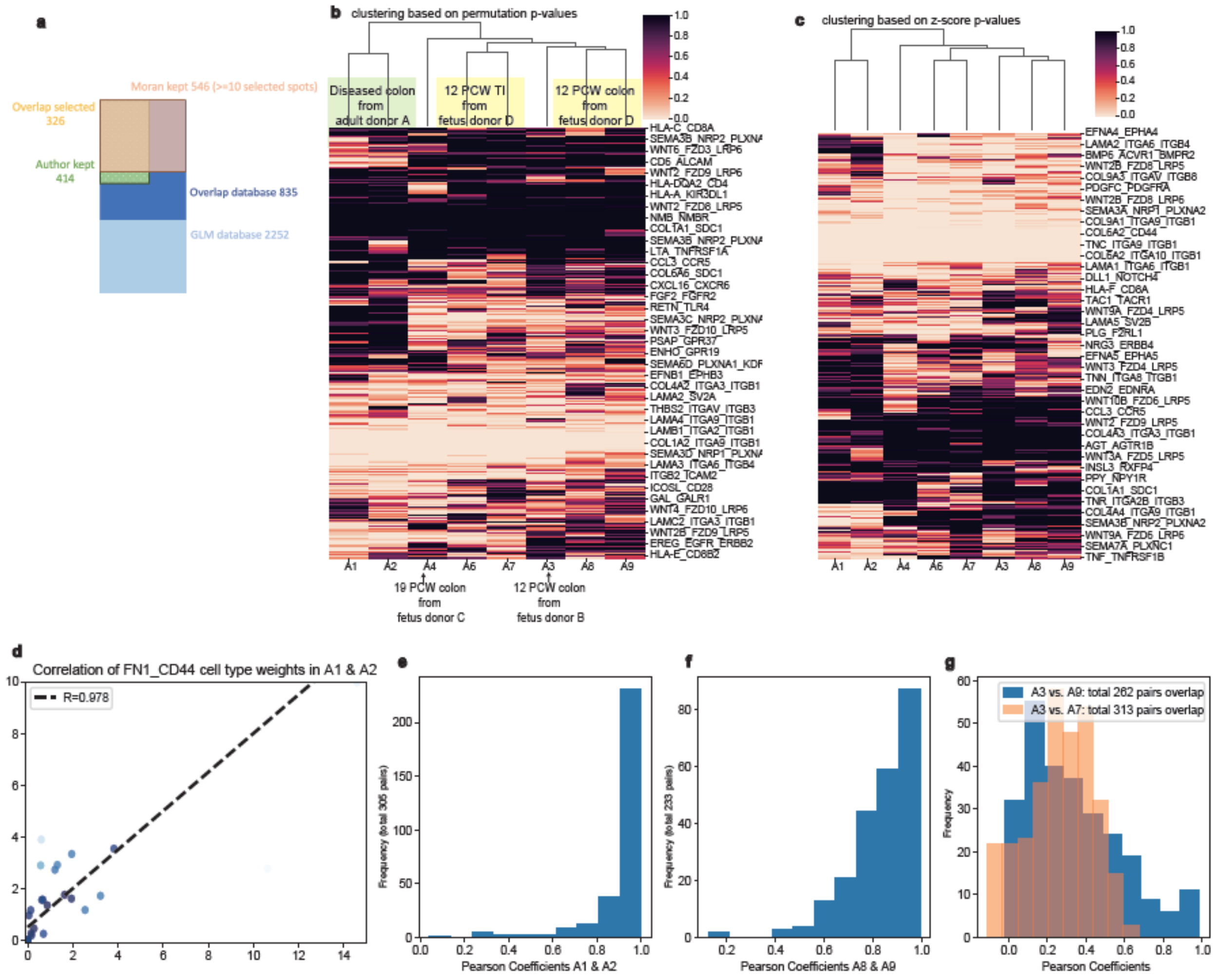

(a)

Supplementary Figure 5: **Global and local selection in the intestine dataset** (a) Comparison of the number of selected pairs across all 8 samples by Corbett, et al. vs. global Moran (permutation approach, $p < 0.05$, one-sided). (b-c) Cluster map based on permutaion (b) or z-score (c) p-values (one-sided). Each row is an interaction; each column is a sample. The sample clustering results were visualised on the top, which were consistent with sample kinship. (d) For FN1_CD44, the correlation of additive cell type weights across selected spots between A1 and A2 (permutation, p <0.1, one-sided). Each dot represents a cell type. x- and y-axis represent additive cell type weights. An Pearson coefficient $R = 0.978$ (two-sided, $p < 1e-16$) was observed. (e-g) Summary coefficient histogram for 4 pairs of samples (i.e. Technical replicates A1 vs. A2 in e, A8 vs. A9 in f, biological replicates A3 vs. A9 and non-biological replicates A3 vs. A7 in g). Each statistic was a Pearson coefficient of additive cell type weight (e.g. a specific example provided in d) computed for an overlapping pair between the samples. The total number of overlapping pairs were specified in y-axis labels or legend.

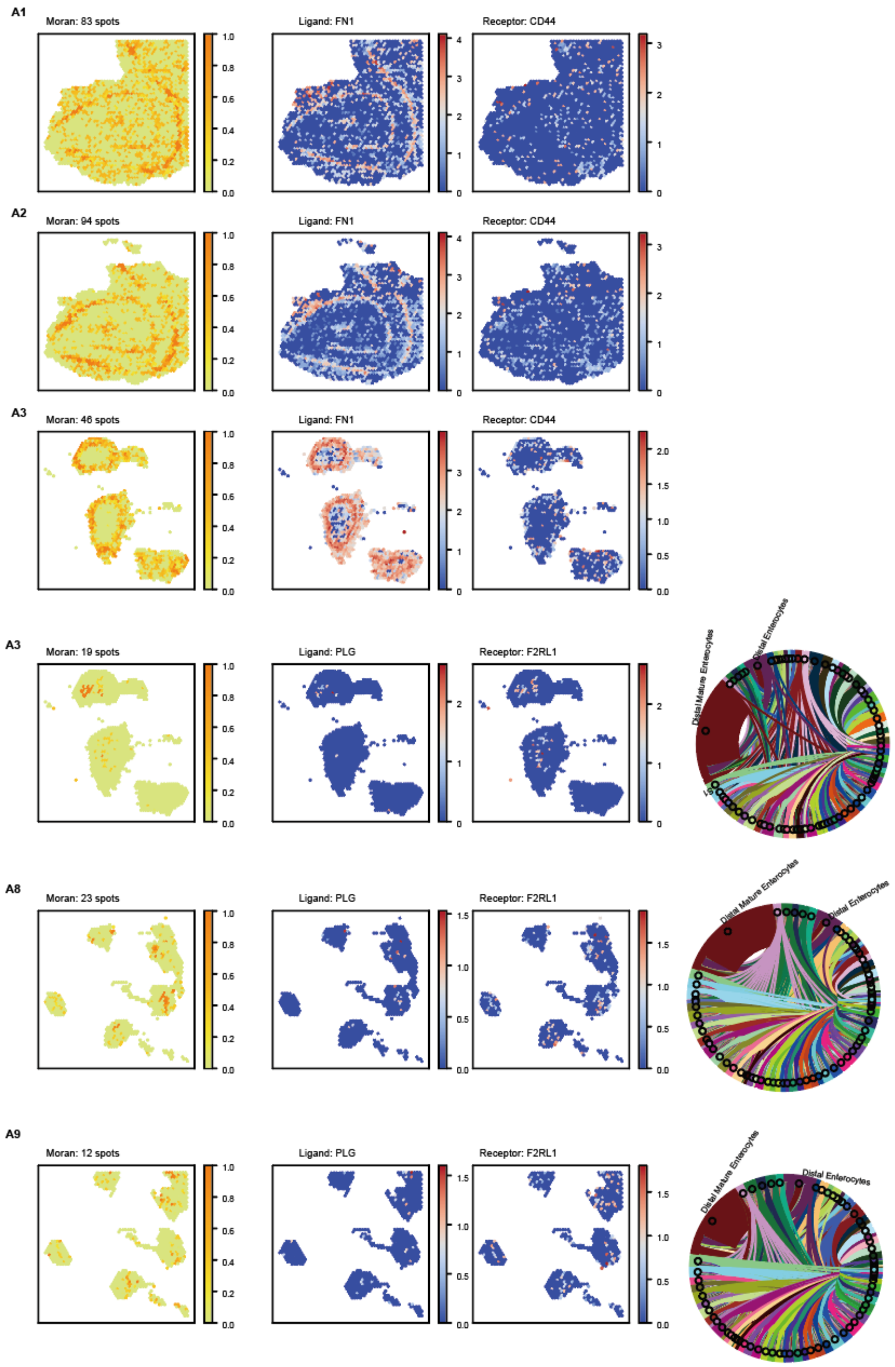

(a)

Supplementary Figure 6: **Spatial plots of FN1_CD44 (Upper) and PLG_F2RL1 (Lower) in the specified samples.** For each lane, local Moran selected spots (z-score approach, coloured by the value of $1-p$), LR expression, and (for PLG_F2RL1) the cell type across selected spots were visualised. In the Moran p-value spatial plots, dot colour indicates 1 - p-values (of all ranges), the selected number of spots refers to only spots with the uncorrected $p < 0.1$. The chord diagram visualises the dominant cell types where edge numbers (or width depending on figure resolution) correspond to weighted cell type composition on selected spots and the edge colour indicates sender cell types. All p-values are one-sided, no adjustments.

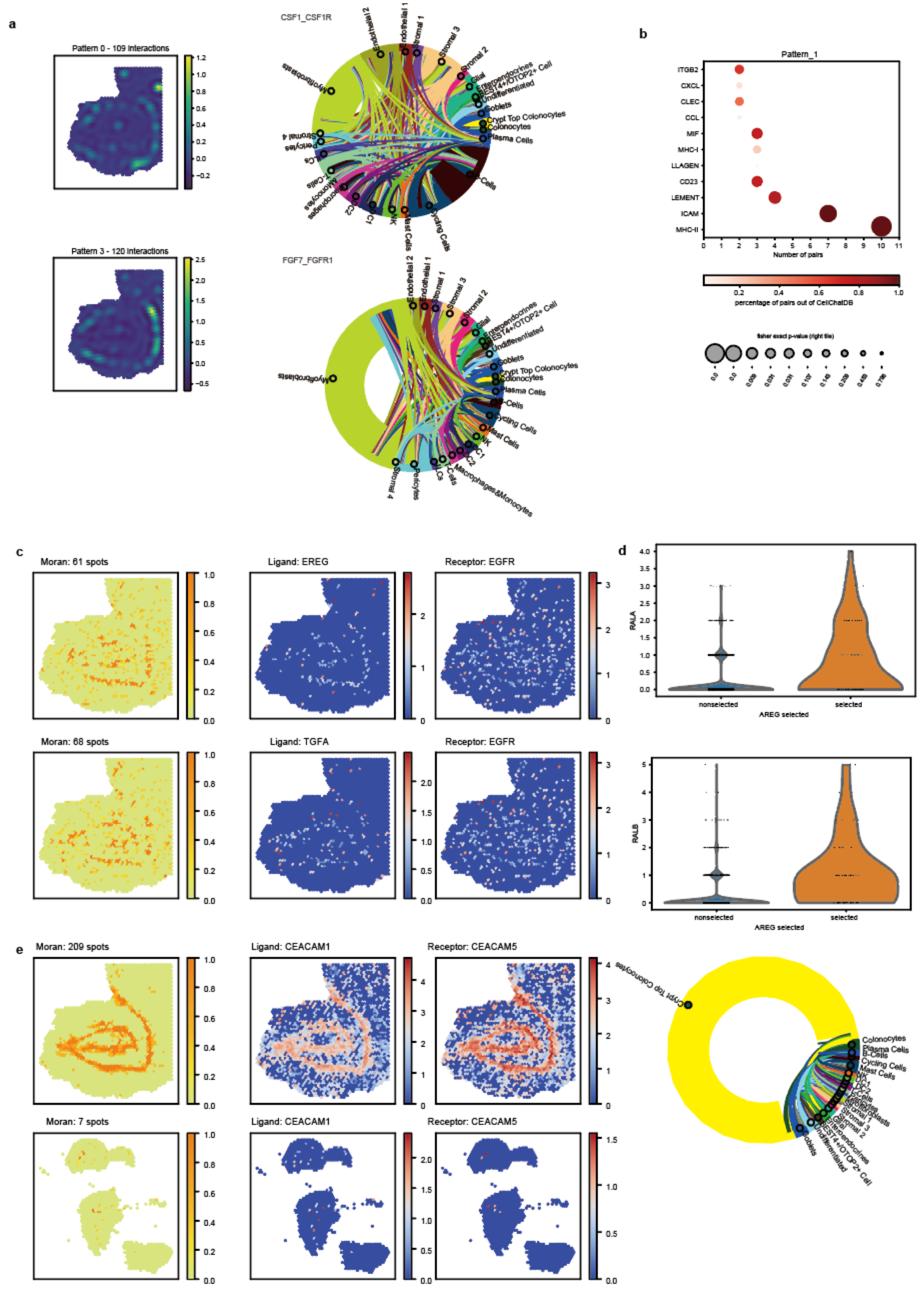

(a)

Supplementary Figure 7: **Local selection results in the adult intestine data** (a) Similar to Fig. 3d, pattern 2 and 3 summary for A1 SpatialDE results. (b) Pathway enrichment results for pattern 0 and 2 (Fisher's Exact Test, one-sided). (c) Similar to Supp Fig 6, two other EGF pathway pairs were visualised in A1, in addition to Fig. 3F. (d) Violin plots of EGF downstream gene expression. (e) The CEACAM1_CEACAM5 interaction pattern visualised in A1 (ubiquitous) versus A3 (sparse). All local p-values are one-sided, no adjustments.

3 Supplementary References

Supplementary References

[1] Daniel A Lim and Arturo Alvarez-Buylla. The adult ventricular–subventricular zone (v-svz) and olfactory bulb (ob) neurogenesis. *Cold Spring Harbor perspectives in biology*, 8(5):a018820, 2016.

[2] Melody P Lun, Matthew B Johnson, Kevin G Broadbelt, Momoko Watanabe, Young-jin Kang, Kevin F Chau, Mark W Springel, Alexandra Malesz, André MM Sousa, Mihovil Pletikos, et al. Spatially heterogeneous choroid plexus transcriptomes encode positional identity and contribute to regional csf production. *Journal of Neuroscience*, 35(12):4903–4916, 2015.

[3] Guillaume Marcy, Louis Foucault, Elodie Babina, Emeric Texeraud, Stefan Zweifel, Christophe Heinrich, Hector Hernandez-Vargas, Carlos Parras, Denis Jabaudon, and Olivier Raineteau. Single cell analysis of the dorsal v-svz reveals differential quiescence of postnatal pallial and subpallial neural stem cells driven by tgfbeta/bmp-signalling. *bioRxiv*, 2022.

[4] Cheuk Ka Tong and Arturo Alvarez-Buylla. Snapshot: adult neurogenesis in the v-svz. *Neuron*, 81(1):220, 2014.
