# Supplementary material for: SpatialDM for rapid identification of spatially co-expressed ligand–receptor and revealing cell–cell communication patterns
Source: Nat Commun. 2023 Jul 6;14:3995. doi: 10.1038/s41467-023-39608-w (PMC10325966; doi:10.1038/s41467-023-39608-w)
Supplement: Supplementary file 2 — Reporting Summary [file 41467_2023_39608_MOESM2_ESM.pdf]

nature portfolio

Corresponding author(s): Dr. Huang Yuanhua

Last updated by author(s): Jun 7, 2023

Reporting Summary

Nature Portfolio wishes to improve the reproducibility of the work that we publish. This form provides structure for consistency and transparency in reporting. For further information on Nature Portfolio policies, see our Editorial Policies and the Editorial Policy Checklist.

Statistics

For all statistical analyses, confirm that the following items are present in the figure legend, table legend, main text, or Methods section.

n/a	Confirmed	
[ ]	[x]	The exact sample size (*n*) for each experimental group/condition, given as a discrete number and unit of measurement
[ ]	[x]	A statement on whether measurements were taken from distinct samples or whether the same sample was measured repeatedly
[ ]	[x]	The statistical test(s) used AND whether they are one- or two-sided<br>*Only common tests should be described solely by name; describe more complex techniques in the Methods section.*
[ ]	[x]	A description of all covariates tested
[ ]	[x]	A description of any assumptions or corrections, such as tests of normality and adjustment for multiple comparisons
[ ]	[x]	A full description of the statistical parameters including central tendency (e.g. means) or other basic estimates (e.g. regression coefficient) AND variation (e.g. standard deviation) or associated estimates of uncertainty (e.g. confidence intervals)
[ ]	[x]	For null hypothesis testing, the test statistic (e.g. *F*, *t*, *r*) with confidence intervals, effect sizes, degrees of freedom and *P* value noted<br>*Give P values as exact values whenever suitable.*
[x]	[ ]	For Bayesian analysis, information on the choice of priors and Markov chain Monte Carlo settings
[x]	[ ]	For hierarchical and complex designs, identification of the appropriate level for tests and full reporting of outcomes
[ ]	[x]	Estimates of effect sizes (e.g. Cohen's *d*, Pearson's *r*), indicating how they were calculated

Our web collection on statistics for biologists contains articles on many of the points above.

Software and code

Policy information about availability of computer code

Data collection	No software was used for data collection, as publically available datasets were used.
Data analysis	The method was developed based on Python (version 3.9.7) and is available at https://github.com/StatBiomed/SpatialDM together with the main analysis notebook.<br>Additional software used was the following: scipy (version 1.7.0),<br>- statsmodels (version 0.12.2),<br>- sklearn (version 0.24.2)<br>- pandas (version 1.3.0)<br>- numpy (version 1.19.5)<br>- threadpoolctl (version 2.2.0)<br>- matplotlib (version 3.4.2)<br>- anndata (version 0.7.6)<br>- scipy (version 1.7.0)<br>- bokeh (version 1.15.2)<br>- holoviews (version 1.15.2)<br>- tqdm (version 4.63.1)<br><br>- SpatialDE (version 1.1.3)<br>- NaiveDE (version 1.2.0)

- RCTD, or Spacexr (version 2.2.0)

For manuscripts utilizing custom algorithms or software that are central to the research but not yet described in published literature, software must be made available to editors and reviewers. We strongly encourage code deposition in a community repository (e.g. GitHub). See the Nature Portfolio guidelines for submitting code & software for further information.

Data

Policy information about availability of data

All manuscripts must include a data availability statement. This statement should provide the following information, where applicable:

- Accession codes, unique identifiers, or web links for publicly available datasets
- A description of any restrictions on data availability
- For clinical datasets or third party data, please ensure that the statement adheres to our policy

All datasets used here are previously published and publicly available:
Raw mRNA counts, log-transformed mRNA counts, and spatial coordinates of the melanoma data were obtained from https://github.com/msto/spatial-datasets;
Raw mRNA counts and spatial coordinates of the intestine data were obtained from https://simmonslab.shinyapps.io/FetalAtlasDataPortal/, GEO: GSE158328 [https://www.ncbi.nlm.nih.gov/geo/query/acc.cgi?acc=GSE158328],
Raw mRNA counts and spatial coordinates of the SVZ data were obtained from https://github.com/CaiGroup/seqFISH-PLUS.
Pre-processed datasets have been deposited at https://figshare.com/s/e62f3537ab56de2ce4ab. For easier reuse, we also included them in the SpatialDM Python package spatialdm.dataset module as follows,
spatialdm.datasets.melanoma()
spatialdm.datasets.intestine(sample = ”A1”)
spatialdm.datasets.SVZ().
The ligand-receptor databases are available from CellChat repository (https://github.com/sqjin/CellChat/tree/master/data).

Human research participants

Policy information about studies involving human research participants and Sex and Gender in Research.

Reporting on sex and gender	N/A
Population characteristics	N/A
Recruitment	N/A
Ethics oversight	N/A

Note that full information on the approval of the study protocol must also be provided in the manuscript.

Field-specific reporting

Please select the one below that is the best fit for your research. If you are not sure, read the appropriate sections before making your selection.

☒ Life sciences ☐ Behavioural & social sciences ☐ Ecological, evolutionary & environmental sciences

For a reference copy of the document with all sections, see nature.com/documents/nr-reporting-summary-flat.pdf

Life sciences study design

All studies must disclose on these points even when the disclosure is negative.

Sample size	We used 3 datasets for demonstrating our methods, with sample sizes on spots ranging from 281 to 2649. For testing differential communication in Fig. 4, we have 2 samples (biological replicates) in adult and 4 samples in foetus.
Data exclusions	No data were excluded from analysis.
Replication	All attempts at replication of data analysis were successful. >=3 replications for real data and simulated data have been performed. No replication of experimental data was performed since we did not collect any experimental data.
Randomization	Randomization usually applies to studies involving participants , cells, or animals. Randomization of samples is not applicable to our study as we do not collect any experimental data.
Blinding	Similar to the reasons above, blinding was not relevant to our study as we report an analysis software as main finding

Reporting for specific materials, systems and methods

We require information from authors about some types of materials, experimental systems and methods used in many studies. Here, indicate whether each material, system or method listed is relevant to your study. If you are not sure if a list item applies to your research, read the appropriate section before selecting a response.

Materials & experimental systems

n/a	Involved in the study	
☒	☐	Antibodies
☒	☐	Eukaryotic cell lines
☒	☐	Palaeontology and archaeology
☒	☐	Animals and other organisms
☒	☐	Clinical data
☒	☐	Dual use research of concern

Methods

n/a	Involved in the study	
☒	☐	ChIP-seq
☒	☐	Flow cytometry
☒	☐	MRI-based neuroimaging
