# Supplementary material for: SpatialDM for rapid identification of spatially co-expressed ligand–receptor and revealing cell–cell communication patterns
Source: Nat Commun. 2023 Jul 6;14:3995. doi: 10.1038/s41467-023-39608-w (PMC10325966; doi:10.1038/s41467-023-39608-w)
Supplement: Supplementary file 12 — Description of Additional Supplementary Files [file 41467_2023_39608_MOESM12_ESM.pdf]

Description of Additional Supplementary Files:

Supplementary Dataset 1: Output from SpatialDM, SpatialCorr, Giotto, CellChat (Trimean & Truncated-mean) and SpaTalk (in separated sheets) in the melanoma dataset.

Supplementary Dataset 2: Output from SpatialDM, SpatialCorr, Giotto, CellChat (Trimean & Truncated-mean) and SpaTalk in the melanoma dataset with 663 unique shuffled pairs to reflect false positive selections.

Supplementary Dataset 3: Full lists of interactions belonging to the 3 clusters in the melanoma dataset (Fig 2b)

Supplementary Dataset 4: GO analysis result for FCER-upregulated spots in the melanoma dataset (Supplementary Figure 3e)

Supplementary Dataset 5: SpatialDM identified results (FDR<0.05) for pairs discussed by Corbett, et al.

Supplementary Dataset 6: SpatialDM results for the 8 samples from the intestine dataset.

Supplementary Dataset 7: Full lists of interactions belonging to the 4 clusters in the intestine dataset (Fig 3d & Supplementary Figure 7a)

Supplementary Dataset 8: Differential analysis for the interactions in the intestine dataset with differential p-values computed by the likelihood-ratio test of global zscores.
